# The role of selective attention in implicit learning: evidence for a contextual cueing effect of task-irrelevant features

**DOI:** 10.1007/s00426-024-02033-9

**Published:** 2024-11-14

**Authors:** Felice Tavera, Hilde Haider

**Affiliations:** Department of Psychology, University of Cologne, Richard-Strauss-Str. 2, 50931 Cologne, Germany

## Abstract

With attentional mechanisms, humans select and de-select information from the environment. But does selective attention modulate implicit learning? We tested whether the implicit acquisition of contingencies between features are modulated by the task-relevance of those features. We implemented the contingencies in a novel variant of the contextual cueing paradigm. In such a visual search task, participants could use non-spatial cues to predict target location, and then had to discriminate target shapes. In Experiment 1, the predictive feature for target location was the shape of the distractors (task-relevant). In Experiment 2, the color feature of distractors (task-irrelevant) cued target location. Results showed that participants learned to predict the target location from both the task-relevant and the task-irrelevant feature. Subsequent testing did not suggest explicit knowledge of the contingencies. For the purpose of further testing the significance of task-relevance in a cue competition situation, in Experiment 3, we provided two redundantly predictive cues, shape (task-relevant) and color (task-irrelevant) simultaneously, and subsequently tested them separately. There were no observed costs of single predictive cues when compared to compound cues. The results were not indicative of overshadowing effects, on the group and individual level, or of reciprocal overshadowing. We conclude that the acquisition of contingencies occurs independently of task-relevance and discuss this finding in the framework of the event coding literature.

## Introduction

In our daily environment, we process information about objects, their shapes, colors, locations, and so on. Thereby, we also register co-occurrences between such features. For instance, imagine your trips to the supermarket: If your favorite pasta comes in a blue package and is always in the same aisle at the supermarket, you will pick it up based on the location and color, without registering more details—you can act routinely in such environments. This kind of learning can occur without any intention to learn and usually, we are also not consciously aware about such learning processes or its contents. Therefore, it is termed implicit learning. It is an important feature of our cognitive system since it helps us to predict future events and thereby to act without effort (Clark, 2013). Another important characteristic of our system is that we learn to discriminate relevant from irrelevant information according to our action goals (Dreisbach & Haider, 2008, 2009; Haider & Frensch, 1996). If we want to buy our supermarket item, we will look for only blue packages, de-selecting other colors. This is a core ability of our attentional system and potentially shapes what we learn from our environment in such everyday actions. The goal of the current study is to ask for the role of selective attention of cues, here manipulated through their task-relevance, in implicit learning processes.

In the field of implicit learning, there has been a long-standing debate about the conditions that are required for such learning processes. When do we notice that certain features of stimuli are co-occurring in a systematic fashion? Do they need to be part of the current action goal or, more broadly, the task-set? Given a confined task context, do features need to be task- or response-relevant to be associatively learned? Or do we encode all the information about all the stimuli of the task at hand in a rather unselective manner and learning occurs automatically whenever the prediction error minimizes due to contingencies inherent in the environment?

### Implicit learning

In the lab, we can study implicit learning processes in several different paradigms, like the serial reaction time task (Nissen & Bullemer, 1987), statistical learning paradigms (Fiser & Aslin, 2001; Reber, 1967), or in contextual cueing paradigms (Chun & Jiang, 1998), to only name a few. The research questions studied with these paradigms are rather similar, yet, research within the different paradigms is usually only loosely connected. Here, we focus mostly on the contextual cueing literature, but integrate also some findings from the other paradigms.

In the original contextual cueing paradigm, participants are instructed to do a visual search task and are asked to find a target letter “T” among a display of distractor letters “L”. For each block throughout an experiment, half of the displays are repeated distractor configurations that consistently predict a target location while the other half of displays are novel configurations. In each trial, participants are asked to report the orientation of the target letter. The contextual cueing effect (CC effect) is defined as a stronger decrease (steeper slope) in response time (RT) for the repeated configurations than for the novel configurations over the course of trials. Note that the configurations are not associated with the orientation of the target, and thus only the contingency between the distractor configuration and the target location can be learned, while the response remains unpredictable. The effect can be traced back to an enhanced efficiency in search, attentional guidance and selection, and, to a lesser extent, to response-related processes (Kobayashi & Ogawa, 2020; Kunar et al., 2007; Schankin & Schubö, 2009, 2010; Sisk et al., 2019). It results in long-term implicit learning effects (Chun & Jiang, 2003). When asked to explicitly discriminate repeated spatial configurations from novel ones, participants are typically not able to do so, and they do not report having learned anything. Therefore, this learning process is assumed to be implicit (Colagiuri & Livesey, 2016; but see Vadillo et al., 2019).

The classical buildup of the contextual cueing task does not seem ideal to study our research question. Because originally, it emphasizes the spatial dimension above all else. When studying the question of the role of task-relevance in implicit learning, we want to compare different cues when they are task-relevant or irrelevant. In the classical contextual cueing, the comparison between different cues would be inherently disbalanced: The predictive feature is the spatial configuration of the distractors, the visual search task is a spatial task, and the requested response is based on the spatial orientation judgement of the target.

Meanwhile, the contextual cueing paradigm has been used in different ways that suggest the possibility of reducing the dominance of the spatial dimension in the task. The empirical evidence supports that, within the paradigm, cues or contexts besides the spatial configuration of distractors are learned, and can guide attention. In the visual domain, multiple studies have shown a CC effect when repeating natural scenes or complex geometric patterns that predict target location, though, involving explicit learning (Brockmole & Henderson, 2006; Brockmole et al., 2006; Ehinger & Brockmole, 2008; Goujon et al., 2012). With more simplistic stimulus material, it has been shown that background color and distractor identity can be implicitly learned to predict the target position. However, when color or shape cues in such form are predictive for target location on top of spatial cues (distractor configuration) being predictive, only spatial cue contingencies are learned, color and shape contingencies are overshadowed (Endo & Takeda, 2004; Kunar et al., 2006, 2013). It has further been shown that spatiotemporal sequences can guide attention (Olson & Chun, 2001), illustrating the wide scope of environmental cues that the cognitive system uses for predictions. So, it seems that a number of features can be used as cues, and probably entirely task-irrelevant features like background color can be learned to predict the target position. Yet, the role of selective attention that might discriminate task-relevant from task-irrelevant stimuli or features, remains unclear in the field of implicit learning.

### Attentional prerequisites for implicit learning

As a cautionary disclaimer: Attention is a widely used and too often under-defined term (Anderson, 2011). Here, we refer to attention as selective attention, not attention as a resource (as in, e.g., Frensch et al., 1998; Nissen & Bullemer, 1987). In the studies we will review here, attention is also mostly operationalized as task-relevance. So, when a stimulus feature is task-relevant, it is considered to be attended, and is consequently integrated into the learning process. This is to be seen separately from the question if the feature is processed consciously or not. In many ways, consciousness and attention are closely related notions (Jiang & Chun, 2003; Mack & Rock, 1998; Tsuchiya & Koch, 2009). It is crucial in the definition of attention to avoid regressive reasoning in the form of invoking a homunculus that fulfils all assumed functions of attention, and is a causal, but unexplained factor in the cognitive system. Therefore, attention in our context is to be understood as the resulting effect when manipulating task-relevance, not as a causal factor on its own. A test for conscious knowledge of the learned contents must be an additional step and is not assumed to perfectly correlate with attending to the to-be-learned features (Tsuchiya & Koch, 2009).

There are two lines of argument with opposing predictions when it comes to attentional prerequisites of implicit learning. The first suggests that task-irrelevant features are not processed in a way that allows for integration into the learning process, either arguing that the features are not processed sufficiently, or that their representational strength is too weak to translate into behavior (Turk-Browne et al., 2005). The second argument suggests that task-irrelevant features are indeed processed to a degree that they can become part of contingencies which then form predictions (Kunar et al., 2013; Miller, 1987).

As to the first line of argument, there are studies that could demonstrate a learning effect only for relevant features. In visual search and also in statistical learning paradigms, participants were instructed to only pay attention to stimuli of one color, and to ignore stimuli of another color (Jiang & Chun, 2001; Jiang & Leung, 2005; Turk-Browne et al., 2005). Because learning of contingencies occurred for the attended color stimuli only, it was concluded that selective attention is a prerequisite for (implicit) learning. Similarly, participants were able to learn a spatial sequence of stimuli, but only additionally learned the contingencies with the identity of these stimuli when they were instructed to count them (Jiménez & Méndez, 1999; Jiménez et al., 1999). Thus, only when the identity of the stimuli were made task- or response-relevant, they were learned (see also Dreisbach & Haider, 2008, 2009). Yet, Jiang and Leung (2005) observed that contingencies in stimuli of an unattended color could be learned in some way, because even though learning did not manifest in behavior at first, it facilitated learning in a subsequent task. In a similar vein, the above mentioned results from Jiang and Chun (2001) cannot be interpreted unambiguously. In their third, higher-powered experiment, they found potential evidence for learning of contingencies also in a task-irrelevant color.

The second group of findings indicate that irrelevant information is also processed and respective learning contents used in future instances. For example, Miller (1987) used a variant of the Eriksen flanker task (Eriksen & Eriksen, 1974). In his experiments, the flankers were not, like originally done in this paradigm, of the same identity as the targets or were otherwise associated with a response. He observed that when these task-irrelevant flankers were associated consistently with a specific response, participants responded faster in these trials compared to when the flanker-response relation was changed. Hence, the irrelevant flankers were associated with the particular response. Similarly, Kunar et al., (2006, 2013) showed that in contextual cueing, task-irrelevant context features such as background color or texture were learned when they were predictive for target location.

An additional finding, however, is that context features like color, texture, or distractor identity are not learned when a spatial configuration is given as an additional cue (Endo & Takeda, 2004; Kunar et al., 2013). This suggests that the spatial configuration could overshadow the learning of other predictive features. This may not be surprising, because, as mentioned above, the task in contextual cueing paradigms inherently emphasizes the spatial dimension. In addition, in the literature on implicit sequence learning, for example, Koch and Hoffmann (2000) suggested that spatial relations of stimuli contributed significantly more to learning effects than other stimulus features. But also generally, the spatial dimension might be distinctly represented in our cognitive system (Mayr, 1996; Paillard, 1991; Schintu et al., 2014). In fact, the spatial dimension might not even be a perceptual feature as such, as it is so tightly bound to the motor system (Gaschler et al., 2012; Goschke & Bolte, 2012; Koch & Hoffmann, 2000; Paillard, 1991).

With respect to findings on attentional mechanisms and learning specifically in the contextual cueing paradigm, these results suggest that their generalizability is strongly limited. The paradigm has, with very few exceptions (Endo & Takeda, 2004; Kunar et al., 2013), not been extended to test other, non-spatial stimulus features. This is particularly a problem when trying to draw conclusions about the learning of task-relevant and task-irrelevant features. Because either spatial features are overshadowing all other visual features (Kunar et al., 2013) because they are weighted more strongly according to the task requirements, or the spatial dimension is represented entirely differently, and thus shows different learning mechanisms than other visual features. Therefore, to conduct a more generalizable test on attentional mechanisms in implicit learning, we designed a novel variant of the task that de-emphasizes the spatial dimension. With this variant, we can contrast the learning of different visual features (color, shape) that are not problematic in terms of the task requirements, or, potentially, their general representation in the cognitive system.

A second point noteworthy in the studies reviewed so far, is that the participants were not able to recognize predictive distractor configurations (Jiang & Chun, 2001; Kunar et al., 2006, 2013) or recall the identity of flankers (Miller, 1987). This was respectively taken as evidence for incidental or implicit learning. However, Vadillo et al (2019) recently questioned the implicit nature of the CC effect, given non-sensitive awareness measures and issues with limited statistical power of many studies in the literature. We will address this with a carefully designed test for conscious awareness, and discuss the issue in light of our results further in the General Discussion.

### Overview of the study

The main goal of the current study was to examine whether selective attending, in terms of task-relevance, is needed to learn the contingencies between distractor features and target location within a contextual cueing paradigm. Importantly, whereas in the original contextual cueing paradigm, the spatial configuration of distractors is the cue for the target location, we implemented nonspatial features of the distractors as cues. We manipulated task-relevance of the predictive cue as following: The shape dimension is task-relevant because the task is to assess the target’s shape (i.e., identity), and thus, the distractor shapes, which are the predictive cues, would be relevant and needed to be processed for the processing of the task. The color dimension, on the other hand, does not appear in any of the task’s processes, neither the search process discriminating distractor from target shapes, nor for the response, that is referred to the distractor shape. The color dimension is thus considered task-irrelevant. A second question concerned cue competition. If more than one feature predicts the target location, will that lead to overshadowing of the task-irrelevant feature, as Kunar et al. (2013) have shown for spatial features? Or are such cue competition effects as overshadowing or blocking the result of explicit or deliberate processes, and thus do not occur in incidental learning paradigms such as our variant of contextual cueing (De Houwer et al., 2005; Schmidt & De Houwer, 2019)? A third question concerned the implicit nature of the acquired contingencies.

In all three experiments, the participants saw spatial configurations of distractors and had to find the target to answer whether a certain characteristic of the target was present. The spatial configurations of the distractors were novel in every trial and did not predict target location. Instead, either the shape (Experiment 1), the color (Experiment 2), or the color and shape (Experiment 3) of the distractors were predictive for the target location.

In Experiment 1, three of six distractor shapes each cued one of four potential target locations whereas for the other three shapes, targets were randomly assigned to the four potential target locations. Note that in this context, shape is a task-relevant cue in so far that it needs to be processed to discriminate the target from the distractor. In Experiment 2, we used the distractor color as a feature to cue the target location. Again, three colors each cued one particular target location, and the other three colors were randomly paired with the target locations. The question here was if the predictive color would be learned as a cue for target location, even though it is neither task- nor response-relevant. To be more precise, color is neither relevant to the search task, as it does not distinguish target and distractors, nor relevant to the response as each color is equally likely to appear with each target identity and there is no color judgement required. In Experiment 3, we tested whether two distinct features of the distractors, shape (task-relevant) and color (task-irrelevant) would be learned to be associated with a target location as a compound, whether both features would be learned independently from one another, or if only one feature would be learned (overshadowing).

## General method

### Stimuli

**Fig. 1:**
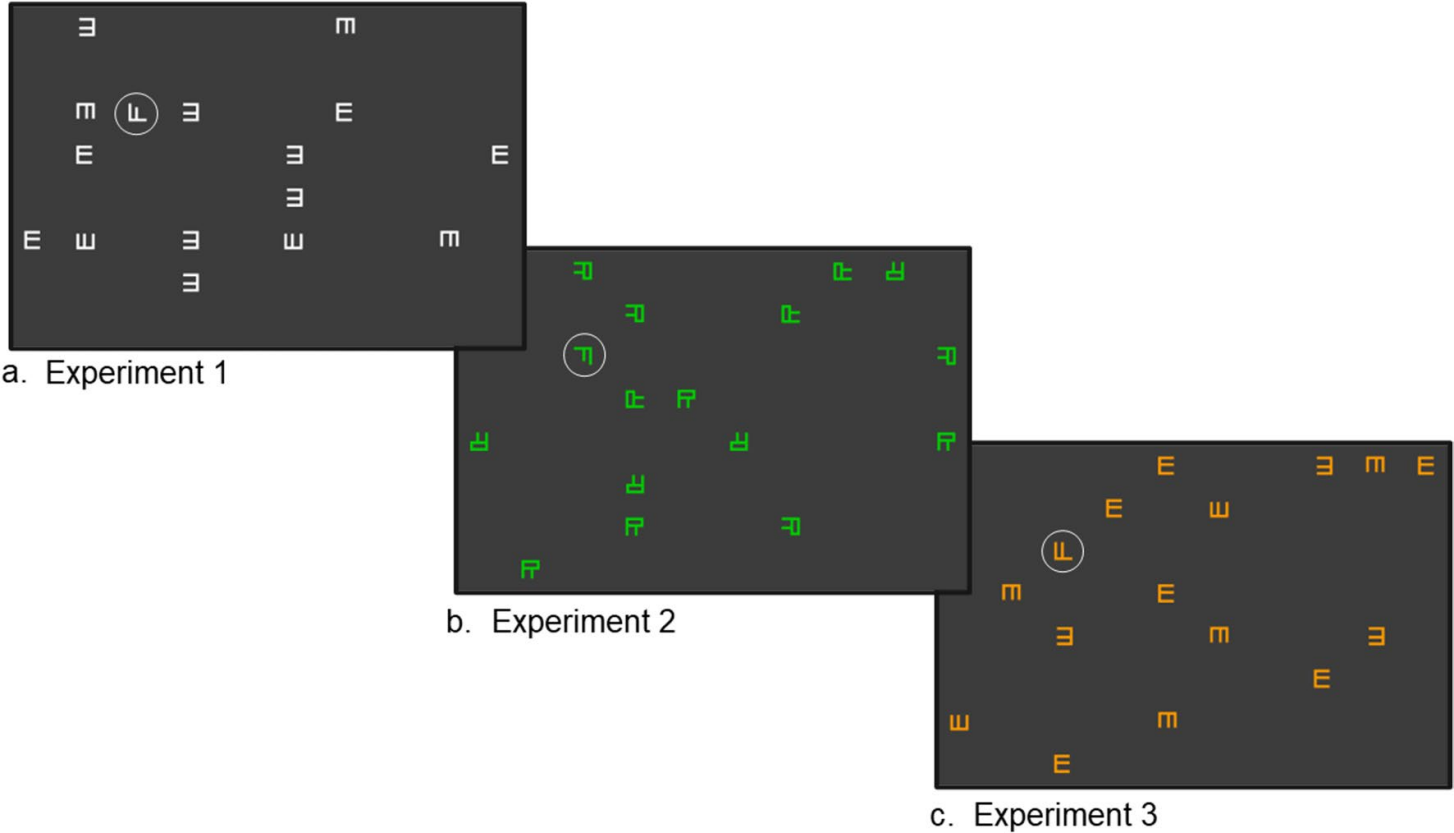
Search Displays in the Training Phase of the Experiments. Note. Exemplary search displays for Experiments 1–3. The target letter “F” is shown in one of four potential target locations. It is circled only for illustration purposes, and was not highlighted in that way in the experiments. **a** An exemplary search display with the E-shaped distractors for Experiment 1 with shape cues. The target letter F has a shorter second horizontal bar. **b** A search display, exemplary green, for color as cue in Experiment 2. **c** A search display with orange E-shapes with the compound cue of color and shape, exemplary for Experiment 3. The target letter F has equally long horizontal bars

The search displays were 15 × 10cm in size, irrespective of the screen size of participant’s computer monitors (see Procedure). The displays were constructed following the method of Bergmann et al. (2019), with minor variations, as described in the following. The displays consisted of 15 distractor letters on a dark grey background (RGB 60, 60, 60) rotated randomly by 0°, 90°, 180°, or 270°. They were organized in a 7 × 10 (invisible) grid, ensuring equal distance between adjacent stimuli, and distributed equally between the two horizontal halves of the display (see Fig. 1). In Experiment 1, all 15 distractor letters of one search display were white (RGB 255, 255, 255) and shaped as one of the six stylized letters A, E, K, P, S, and W. In Experiment 2, all 15 distractor letters of one search display were R-shaped and were colored in the six colors green (RGB 1, 204, 0), orange (RGB 254, 153, 0), blue (RGB 0, 0, 254), red (RGB 254, 0, 0), pink (RGB 255, 0, 254), and cyan (RGB 1, 255, 255). In Experiment 3, the 15 distractor letters were distinctly shaped and colored, with each color matched to one shape (for example, S shaped distractors were always colored in pink, and so on). Colors were those of Experiment 2 and shapes those of Experiment 1. The color-shape matching was permuted across participants.

**Fig. 2:**
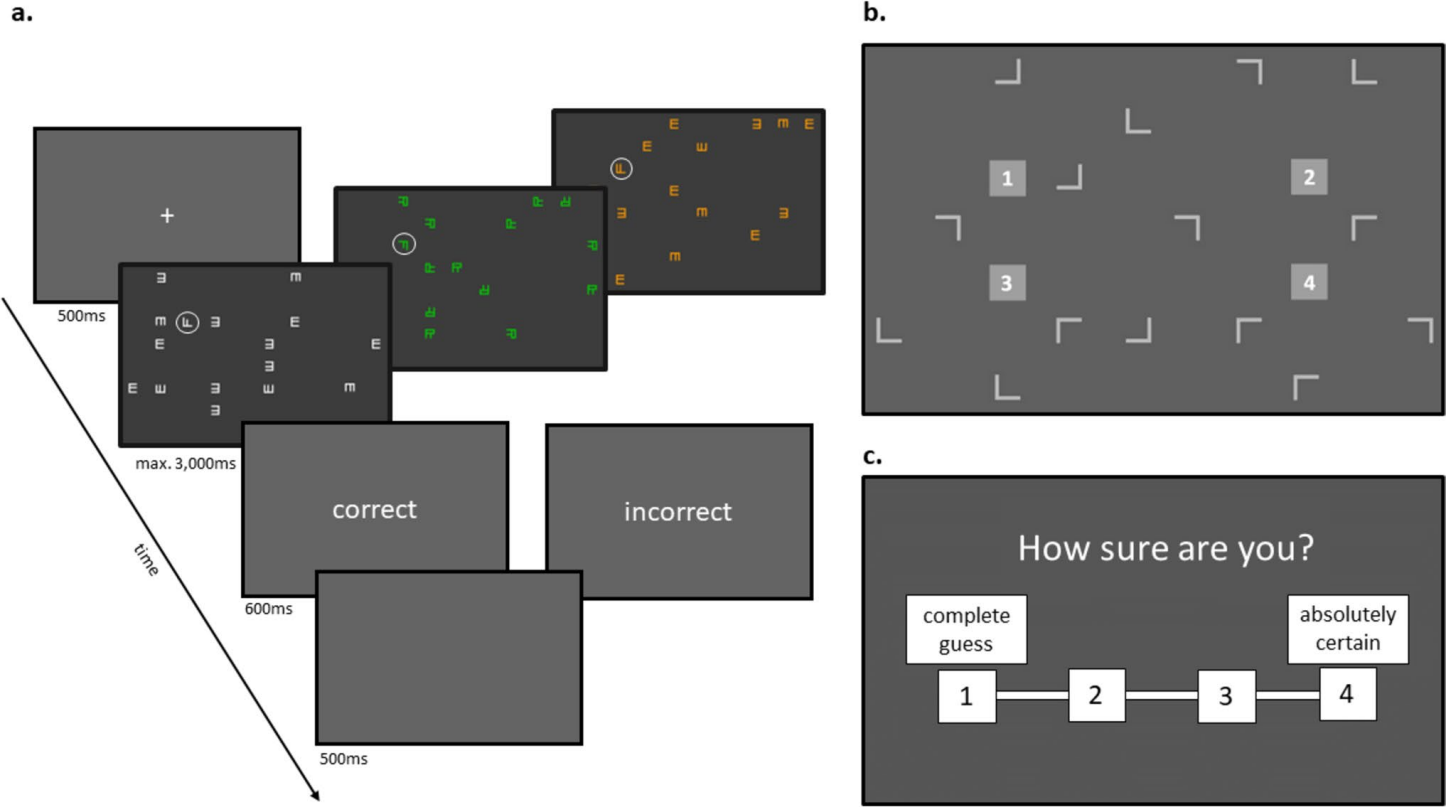
Procedure for Experiments 1–3. Note. **a** The structure of a training trial. **b** An example of a display in the generation task in which the four possible target locations are marked with the numbers 1–4. **c** The 4-level confidence scale

In one of four possible target locations (see Fig. 2b), there was either an F with equally long horizontal bars, or an F with a shorter second horizontal bar as target letter (see Fig. 1). All targets were randomly rotated to the right (90°) or the left (270°).

For half of the cues, contingencies between distractor characteristic and target location were fix. That is, three of the distractors’ colors or shapes or shape/color combinations were always paired with one respective target locations: For example, for Experiment 1, three shapes were each 100% contingent with a target location. The shape-target location matching was permuted across participants. For the other three letters, the four target locations were equally likely. In Experiment 2, the same applies for color-target location matchings.

### Procedure

All three experiments were conducted in accordance with the Declaration of Helsinki. Participants were recruited online via Prolific and were reimbursed according to Prolific’s “ethical reward” standards. They were redirected to Pavlovia, where the experiments were uploaded from PsychoPy2 (version 2020.2.4; Peirce et al., 2019) and adapted to the JavaScript environment. Participants were informed about the procedure of the experiment and asked to give their informed consent.

Participants were first asked to follow a screen scaling procedure (Wakefield Morys-Carter, 2021). With the arrow keys on their keyboard, they were asked to adjust an image of a credit card on the screen to the size of an actual bank or credit card. This procedure ensured equal size of the search displays for every participant irrespective of the monitor size or aspect ratio.

All three experiments consisted of three main parts: A short practicing phase, a training phase, and lastly, a generation task. In the first 12 practice trials, they were shown displays with white, L-shaped distractor letters to get used to the task for the training. In each trial of the training (see Fig. 2a), a fixation cross was presented for 500ms. Then, the search display appeared for a maximum of 3000ms or until the response. The response window started with the appearance of the search display and lasted 4000ms. Participants were instructed to search for the target letter “F” among distractor letters, and to identify if the second bar was short or long, respectively pressing the “S” or “L” key on their keyboard with their index fingers as quickly and accurately as possible. The trial ended with a feedback text (“correct” or “incorrect”) that appeared on the screen for 600ms and was followed by a blank inter-trial-interval of 500ms. The training consisted of 15 blocks of 48 trials each. Participants were given the opportunity to take a short self-paced break after every block.

After training, a so-called generation task (Chun & Jiang, 2003) started to assess participants’ awareness about the cue-target location contingency. The generation task contained one block of 48 trials. It was designed such that participants were provided with a similar retrieval context as in the learning environment, as participants were shown the search displays of the training phase. This similarity of the environment and task during training and test phases provide similar sensitivities of both tests, thus increasing the chance to detect potential conscious knowledge (Shanks & St. John, 1994). In the test phase, participants were presented with search displays of the training phase, just that there was no target letter, but instead, the four potential target locations were marked with the numbers 1–4 (see Fig. 2b). Participants were instructed to indicate in which target location they think the target letter was presented using the number keys on their keyboard. Afterwards, a visual scale from 1 (labeled “complete guess”) to 4 (labeled “absolutely certain”) appeared on the screen (see Fig. 2c), and participants were asked to indicate their confidence with their generation response, again using the number keys on their keyboard.

To finish the study, participants lastly were redirected to Qualtrics (Qualtrics, 2020) or SoSci Survey (Leiner, 2024) to respond to some questions about the experiment. They were asked to report technical issues, their ideas on the purpose of the study or if they noticed anything, if and why the task became more difficult or easier, and if they had noticed any regularities or contingencies.

*General data analysis.* The statistical analysis was conducted in R Statistical Software (version 4.1.0; R Core Team, 2021). We used the dplyr package for most data manipulation (Wickham et al., 2023), the lme4 package for fitting models (REML; Bates et al., 2014) with restricted maximum likelihood (REML) model fit, and the lmerTest package (Kuznetsova et al., 2017) with the Satterthwaite's method for *t*-tests. Note that for the *χ*2 tests for model comparisons, the models are refitted using maximum likelihood (ML). Graphs were created with the ggplot2 package (Wickham 2016). The study’s design and analysis were not pre-registered. Data and analysis scripts are available on OSF.

For the training, we excluded incorrect trials, and trials with the one target location out of the four that appeared less frequently. Because there were three cues, that is, three colors, shapes, or color/shape compounds, matched to one target location, and the other three colors or shapes were equally often paired with all four target locations, one target location is consequentially never predicted by a cue, and is also less frequent than the other three target locations.^1^ In trials with the less frequent target location, we would obtain significantly longer RTs, because of the common probability cueing effect (Golan & Lamy, 2024). This is why, for the RT analysis, we excluded trials with the less frequent target location, and only compared predictive and unpredictive trials for the three equally frequent target locations. Further, to account for the intertrial priming effect (Golan & Lamy, 2024; Kabata & Matsumoto, 2012), that is, shorter RTs for trials in which the target location is repeated from trial $n-1$, we excluded such target location repetition trials as well. Because of those necessities to exclude trials based on the design, we decided not to exclude any more trials based on outlier analysis. This is also in line with recent analyses that outlier exclusion procedures for RT analyses might add biases and power issues, and thus do more harm than good (Miller, 2023).

For the RT analyses, we fitted a mixed-effects model for two reasons. First, the method does not require aggregating data from multiple blocks into epochs, and thus less data is summarized (for a similar analysis, see e.g., Bergmann et al., 2019). Second, with a mixed-effects model, we are able to account for the repeated-measures design more efficiently by including subject as random effect (Huta, 2014; Weinfurt, 2000). It should be noted that conducting power analyses with mixed-effects models is a challenge due to the complexity of parameter and variance estimation, particularly with unknown random effect structures and their interactions (West et al., 2022). Because we had no prior data from our paradigm to obtain such estimates, we chose to refrain from conducting a power analysis. Yet, the sample sizes and number of observations in all three experiments are larger than the recommended minimum for mixed-effects model analyses (Hox et al., 2017).

We selected a model with two fixed effects: context (predictive or unpredictive color/shape/color-shape compound) and block (as time variable) as factors. Context as a dichotomous factor was coded with contrasts − 0.5 and 0.5, and the block factor was coded with block-1 for better interpretability. Our fixed effects were deduced from theoretical considerations, and on top of that, tested in model fits, but we decided on random effects solely based on the data. The argument here is that, on the one hand, it has been argued that maximal models are best for keeping the Type I error low while at the same time not significantly decreasing statistical power (Barr et al., 2018). On the other hand, however, simulations have shown that the statistical power to detect significant fixed effects can in fact be increased when opting for a random effect structure that fits the data better, as compared to implementing the full model (and more so for complex models; Matuschek et al., 2017; for a similar assessment of model selection in repeated-measures designs see also Stroup, 2013). To balance the Type I error rates and statistical power, Matuschek et al. (2017) suggest to select a model based on a selection criterion such as the Akaike information criterion (AIC; Akaike, 1998) or the Bayes information criterion (BIC; Schwarz, 1978) that assess goodness-of-fit. We decided to follow this line of argument to identify the most parsimonious model while balancing the Type I error and power. Thus, we compared various potential random effect structures based on the AIC, and additionally provide *χ*^2^ significance tests of log-likelihood (-2LL) changes from nested models. The results from all potential random effect structure models and model comparisons are accessible via the analysis script uploaded to OSF.

Following up on results from frequentist *t*-tests, we report Bayes factors that additionally indicate the strength of evidence for the null or alternative hypothesis. We computed Bayes factors (BF) using JASP (JASP Team, 2022) with the JASP default priors for *t*-tests (following Morey & Rouder, 2022; Cauchy distribution with a width of *r* = 0.707). The semantic labels for BF interpretation are taken from Jarosz and Wiley (2014).

For the generation trials, we tested the objective performance (target placement) against chance level (25% because of four response alternatives). Because one of the four target locations is far less frequent than the other three, one could even postulate, that chance level is rather 33%, as if it was choosing between the three more frequent target locations. Still, we opted for the more conservative approach to test against 25%. For the analysis, we selected only the predictive shapes and colors, because (implicit) knowledge about cue and target location associations could only be acquired for those. To assess conscious awareness of the learning contents, we followed the “consciousness-selectivity” argument of Michel (2023), proposing that differences in consciousness lead to differences in metacognitive efficiency, distinguishing correct from incorrect responses (for a similar method see Persaud & McLeod, 2008). To assess the relationship between the objective performance measure and the subjective confidence measure, we computed variables for relative frequencies of correct response under the condition of high confidence (correct|high) and low confidence (correct|low). The logic here is that participants with explicit knowledge of a pairing of cue and target location should be able to make a metacognitive assessment of their knowledge (Haider et al., 2011). Thus, when knowing a pairing explicitly, their response should be correct, and their confidence should be high. Note that we summarized confidence ratings of 1 and 2 as low, and ratings of 3 and 4 as high confidence, taking a more conservative approach that considers individual response biases and regression to the mean (both would potentially result in an avoidance of the extreme scale values).

## Experiment 1

In the first experiment, as a way to introduce our variant of the contextual cueing paradigm, we were using distractor identity features (i.e., shape) as cues, instead of the original spatial configuration context cue. We tested whether different shapes can be learned to predict target location, and thus facilitate and speed visual search processes. First, we constructed the task so that three of the six possible shapes of distractors were 100% predictive of target location.

### Method

#### Participants

30 participants were recruited via *Prolific* (15 female, 1 diverse; *M*_age_ = 41.37; *SD*_age_ = 13.50). Participants were prescreened for living in the UK (participation took place during daytime for all participants), being fluent in English, have normal or corrected-to-normal vision, and had not taken part in a previous contextual cueing experiment of our lab.

### Results

#### Training

**Fig. 3:**
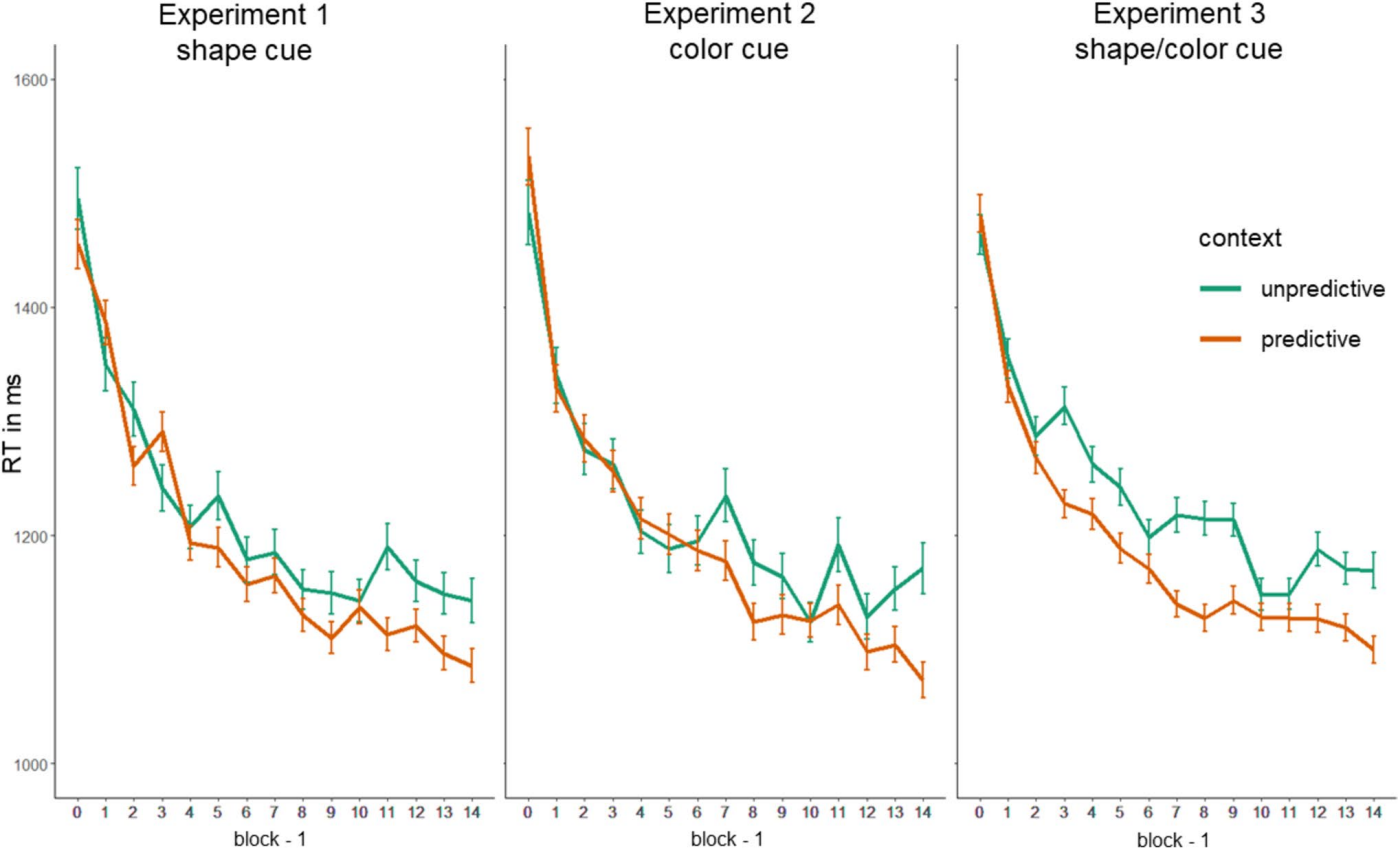
Response Times in Milliseconds by Block and Context (Predictive/Unpredictive). Note. Error bars indicate standard errors

The removal of incorrect responses, repeated target location trials, and trials with the less frequent target location resulted in a 21.59% trimming. Mean accuracy was 95.51% (*SD* = 0.21), mean RT for the cleaned data set was 1193.73ms (*SD* = 436.37). RTs over the course of the blocks, separated by predictive and unpredictive context, are shown in Fig. 3.

**Fig. 4:**
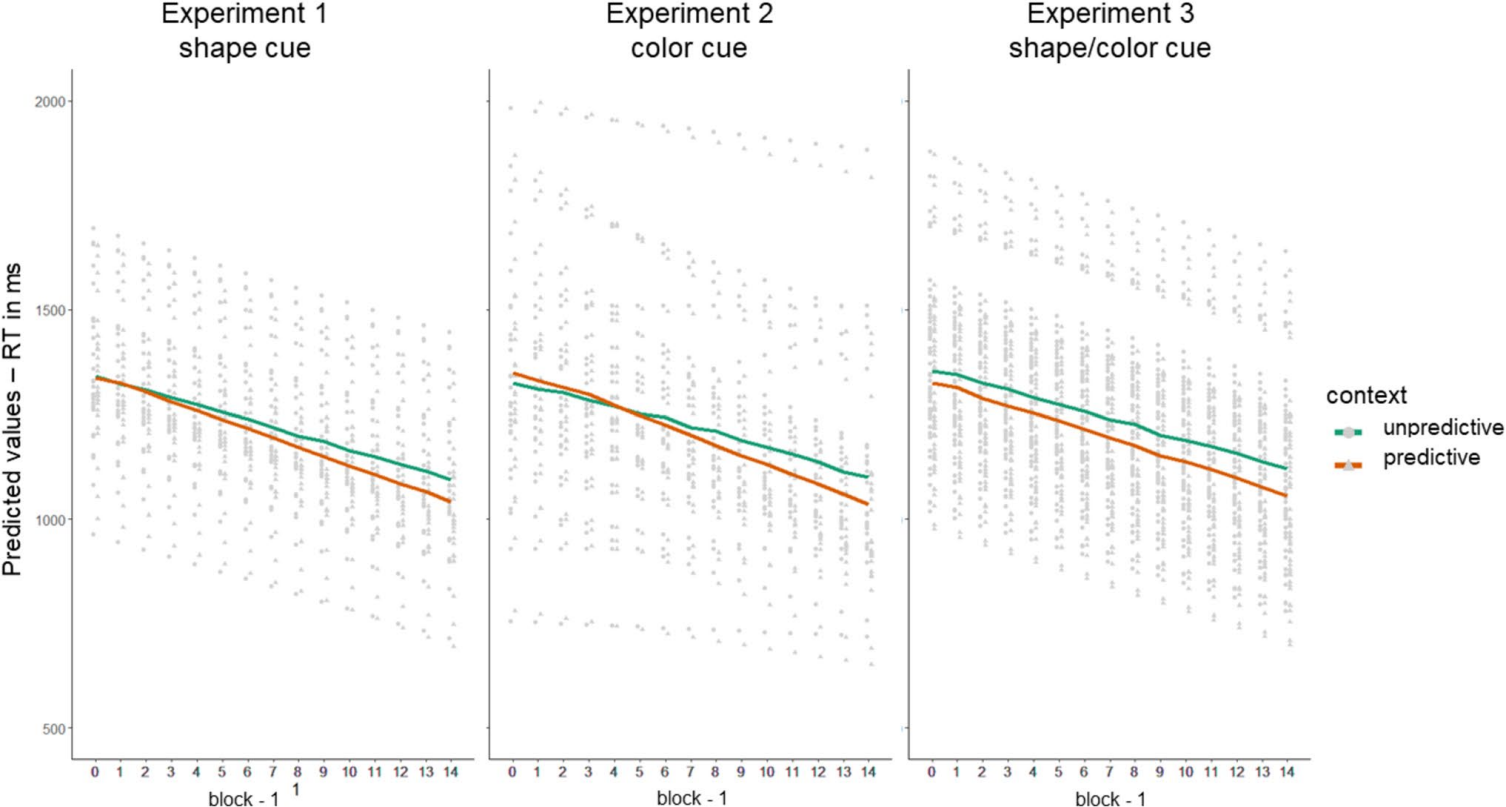
Predicted Response Times in Milliseconds Per Participant, Block and Context (Predictive/Unpredictive) As Predicted by Mixed Effects Models for Experiments 1–3. Note. The light grey points indicate the individual intercepts/slopes for participants, the dot and triangle indicate the difference between predictive and unpredictive contexts. The thick lines indicate the overall effect of context

For RT as dependent variable, we first tested whether the fixed effect structure hypothesizing an interaction of context and block was the best fit for the data. A comparison of AICs of models with no random effect structure and no fixed effect (AIC = 254,040.9), only block as fixed effect (AIC = 253,372.1), an additive (AIC = 253,359.2), and an interaction effect (AIC = 253,354.7), confirmed that the interaction term model indeed yielded the best fit. In a next step, we compared random effect structures. Allowing for random slopes for context across participants (AIC = 250,835) yielded a better fit than only random intercept for participants (AIC = 250,864; *χ*^2^(2) = 32.287, *p* < 0.001). Other and more complex random effect structure models did not converge or produced a singular fit. In the random slopes and random intercept model, the fixed effects context and block, as well as their interaction, were significant (see Table 1 and Fig. 4).

**Table 1:** Response time analysis results for experiments 1–3

	Estimate	SE b	95% CI b		df	T	*p*
			LL	UL			
Experiment 1 (shape)							
Intercept	1345.15	30.98	1284.42	1405.88	43	43.415	< .001***
Context (pred)	3.01	15.11	− 26.60	32.63	94	0.199	.842
Block	− 19.81	0.72	− 21.21	− 18.41	16,878	− 27.703	< .001***
Context x Block	− 4.07	1.43	− 6.87	− 1.26	16,886	− 2.845	.004**
Experiment 2 (color)							
Intercept	1351.26	51.20	1250.92	1451.61	28	26.40	< .001***
Context (pred)	25.60	11.91	2.26	48.95	16,436	2.150	.032*
Block	− 19.91	2.56	− 24.93	− 14.88	28	− 7.766	< .001***
Context × Block	− 6.65	1.44	− 9.47	− 3.83	16,436	− 4.621	< .001***
Experiment 3 (compound)							
Intercept	1347.36	27.09	1294.27	1400.46	59	49.740	< .001***
Context (pred)	− 26.87	12.52	− 51.41	− 2.34	141	− 2.147	.032*
Block	− 18.47	0.54	− 19.52	− 17.41	32,194	− 34.45	< .001***
Context × Block	− 2.68	1.07	− 4.78	− 0.57	32,196	− 2.496	.013*
Experiment 3 (single cue blocks)							
Intercept	1118.51	26.82	1065.94	1171.08	57	41.710	< .001***
Context (pred)	− 32.51	11.42	− 54.90	− 10.11	4491	− 2.846	.004*

*SE* standard error, *CI* confidence interval, *LL *lower limit, *UL *upper limit. Mixed-effects model computed coefficient, standard error and confidence interval for the coefficient, degrees of freedom, *t*-Avalue, and *p*-value are displayed for each predictor and experiment. Degrees of freedom are rounded

***$$p\le .001$$, ** $$p\le .01$$, *$$p\le .05\text{A}$$

#### Generation task

Overall accuracy in the generation task for the predictive contexts was 27.22% (*SD* = 0.45) which was not significantly above chance level, *t*(29) = 1.138, *p* = 0.132. Mean confidence rating (1–4 scale) was 1.64 (*SD* = 0.50) for the predictive shapes, and 1.59 (SD = 0.51) for unpredictive shapes, which was not significantly different in a paired, one-tailed *t*-test, *t*(29) = -1.244, *p* = 0.112 (*BF*_01_ = 1.452; interpreted as anecdotal evidence for the null hypothesis). We pooled confidence ratings for predictive shapes of 1 (*n* = 364) and 2 (*n* = 267) as low, and ratings of 3 (*n* = 70) and 4 (*n* = 19) as high confidence. The difference between relative frequency of correct|high (23.66%) and correct|low (25.62%) responses was not significant in a paired, one-tailed *t*-test, *t*(29) = -0.317, *p* = 0.623 (*BF*_01_ = 6.439; interpreted as substantial evidence for the null hypothesis).^2^ A Pearson’s product-moment correlation test showed a significant correlation within participants, between accuracy in the generation task and the CC effect, in the following simply defined as predictive – unpredictive RT, *r*(28) = 0.367, *p* = 0.047, but no correlation between accuracy and confidence in the generation task, *r*(28) = 0.238, *p* = 0.206.

### Discussion

In Experiment 1, we could show that participants learn to predict the target location from the shapes of distractors. The interaction of context and block was significant, due to steeper RT slopes for predictive shapes than for unpredictive shapes. This search speed advantage for predictive shapes develops over the course of blocks, indicating learning to use the cues to find the target. The regression coefficient of the interaction of context and block indicates an RT difference increase between predictive and unpredictive contexts by -4.07ms with every block. The significant main effect for block indicates that there is also a general training effect. Experiment 1 thus showed that a task-relevant visual feature that characterizes distractor identity can be learned to cue the target location. We do not find a main effect of context, which is to be explained by the slowly emerging learning such that the RT difference between contexts is not consistent but only present in roughly the second half of the training phase.

As indicated by a lack of relation between objective measure (the generation task) and the confidence measure, we would argue that the knowledge of contingencies between cue and target location remained implicit.

## Experiment 2

In Experiment 1, we established a novel variant of the contextual cueing task with a non-spatial but task-relevant feature. Participants had to process the respective shape in order to find the target letter F. In the second experiment, we tested if participants even learned that different colors of the distractors predicted different target locations. As argued above, color is an entirely task-irrelevant feature.

### Method

#### Participants

Thirty participants were recruited via *Prolific* (19 female; *M*_age_ = 40.13; *SD*_age_ = 12.20). One participant was excluded from analysis due to poor performance in the training (48.88% accuracy). Participants were prescreened for living in the UK (to ensure that it was daytime), being fluent in English, having normal or corrected-to-normal vision, and not having taken part in a previous contextual cueing experiment of our lab.

### Results

#### Training

Incorrect responses, trials with the less frequent target location or target location repetition were excluded from analysis (23.63% trimming). Mean accuracy was 94.49% (*SD* = 0.23), mean RT in the cleaned data set was 1205.86ms (*SD* = 470.25). Mean RTs over the course of the blocks, separated by predictive and unpredictive context are displayed in Fig. 3.

For RT as dependent variable, we first tested, whether the fixed effects structure hypothesizing an interaction of context and block, was the best fit for the data. Comparing AICs of models with no random effect structure and no fixed effect (AIC = 249,809.7), only block as fixed effect (AIC = 249,243.1), an additive (AIC = 249,237.2), and an interaction effect (AIC = 249,223.6), revealed that the interaction term model yielded the best fit. In a next step, we compared random effect structures. Allowing for random slopes for the factor context (AIC = 244,395) was not a better fit than the random intercept model (AIC = 244,394; *χ*^2^(2) = 3.642, *p* = 0.162), but random slopes for the factor block fit significantly better in comparison to the random intercept model (AIC = 244,144;; *χ*^2^(2) = 243.1, *p* < 0.001). More complex models did not converge or produced a singular fit. In the random slopes and random intercept model, the fixed effects context and block, as well as their interaction, were significant (see Table 1 and Fig. 4).

#### Generation task

Overall accuracy for the predictive colors was 30.03% (*SD* = 0.46) which was significantly above chance level, *t*(28) = 1.83, *p* = 0.039, *d* = -0.34, *BF*_10_ = 1.639 (interpreted as anecdotal evidence for the alternative hypothesis). Mean confidence rating (1–4 scale) was 1.52 (*SD* = 0.72) for the predictive colors and 1.52 (SD = 0.71) for unpredictive colors, which was not significantly different in a paired, one-tailed t-test, *t*(28) = 0.041, *p* = 0.516 (*BF*_01_ = 5.225; interpreted as substantial evidence for the null hypothesis). We summarized confidence ratings of predictive colors of 1 (*n* = 414) and 2 (*n* = 216) as low, and ratings of 3 (*n* = 52) and 4 (*n* = 14) as high confidence. The difference between relative frequency of correct|high (19.78%) and correct|low (29.49%) responses was not significant in a paired, one-tailed t test, *t*(28) = -1.66, *p* = 0.946 (*BF*_01_ = 12.268; interpreted as strong evidence for the null hypothesis). A Pearson’s product-moment correlation test showed no significant correlation between accuracy in the generation task and CC effect, *r*(27) = -0.17, *p* = 0.385, and no correlation between accuracy and confidence in the generation task, *r*(27) = 0.22, *p* = 0.245.

### Discussion

As in Experiment 1, the significant interaction between context and block indicates learning of the cue and target location association. Also here, the RT slope for predictive colors is steeper than for unpredictive colors across training blocks. Other than in Experiment 1, we also obtain a significant main effect of context in the opposite direction as hypothesized (predictive contexts are slower than unpredictive contexts overall). As can be seen from Fig. 4, this finding is based on the reverse effect in the first block of the training phase. This can be demonstrated when, in a post-hoc analysis, we exclude the first block and compute the model (however, with only random intercepts as the random slope model did not converge). Then, the main effect of context is no longer significant, *b* = 17.60, *p* = 0.181, but block, *b* = -14.56, *p* < 0.001, and the interaction effect is still significant, *b* = -5.84, *p* < 0.001. Testing the same model, but with context and block as additive factors, both main effects are significantly negative, context, *b* = -26.47, *p* < 0.001, and block, *b* = -15.03, *p* < 0.001. Both models fitted to the data with the first block removed, with additive and interaction effect respectively, are in line with a context learning effect. When not accounting for the interaction, both main effects are negative, which means that with block number, RTs decrease significantly, and for context, that RTs in predictive contexts are significantly shorter than in unpredictive contexts. In our original models, the main effect for context is overlaid by an interaction effect that stems from the first block. This interaction is however still significant when removing the first block. This significant interaction effect of context and block is consistent with the pattern of a context learning effect across blocks.

Building on previous research that has assigned task-relevance a major role in implicit learning (Jiang & Chun, 2001; Jiang & Leung, 2005; Jiménez & Méndez, 1999; Turk-Browne et al., 2005), color contingencies with target location should not have been learned. Nevertheless, we observe an RT advantage for predictive versus unpredictive colors over the course of the blocks that indicate learning. The model predicts a learning effect, which manifests in the RT difference between predictive and unpredictive contexts that increases by -6.65ms with every block.

In the generation task, we find a performance that is significantly above chance level. First of all, according to the Bayes Factor, there is no strong evidence for the hypothesis that participants are indeed better than chance level. Secondly, we do not exclude the possibility of a higher-than-chance performance even under the premise of implicit learning. There is the chance that, given the training and the test phase are so similar, implicit knowledge spills over to the test phase, and causes higher performance (Michel, 2023, but see Shanks & St. John, 1994). However, the awareness test lies in the association with a contingency measure, as this provides a metacognitive judgement. Here, we find no evidence of higher confidence in correct responses, which would be expected if participants had a metacognitive awareness of their knowledge of the contingencies, making it explicit knowledge (Dienes & Seth, 2010).

## Experiment 3

In this experiment, we aimed to test the effects of cue competition. The task and set-up are the same as in Experiments 1 and 2, but here, we provided distractors that were characterized by a one-to-one mapped shape and color. Shape and color redundantly predicted target location. So, both, independently or integrated (as a compound or one overshadowing the other), could be learned to be used as a cue for target location. To test this question, additionally to the 15 training blocks, two blocks with 48 trials each were implemented. The distractors here contained either only color but not shape information (colored “R” distractors), or only shape but not color information (white distractors in six shapes) respectively. It was counterbalanced between participants if the first of the two blocks was the color or the shape block. With these blocks, we were able to test whether the learning effects for each individual cue would be additive, indicating independent learning effects, or underadditive, indicating compound learning or overshadowing effects.

### Method

#### Participants

For Experiment 3, the number of participants was doubled relative to Experiments 1 and 2. This aimed to increase statistical power when testing effects in the single cue blocks, as there were only 48 trials per participant and cue. An increase in the number of participants was preferred to an increase in the number of trials in the single cue blocks, because of the risk of participants learning the contingencies anew. Thus, 60 participants were recruited via *Prolific* (40 female; *M*_age_ = 39.29; *SD*_age_ = 11.98). They were prescreened for living in the UK, being fluent in English, have normal or corrected-to-normal vision, and had not taken part in a previous contextual cueing experiment of our lab. Two participants were excluded due to poor performance in the training phase (49.02% and 51.84% accuracy respectively).

### Results

#### Training

Incorrect responses, target location repetitions and the infrequent target position trials were excluded (22.62% trimming). Mean accuracy was 95.06% (*SD* = 0.22), mean RT in the cleaned data 1196.71ms (*SD* = 463.97). Figure 3 displays mean RTs over the course of the blocks, separated by predictive and unpredictive context. For the fixed effect structure, comparing AICs of models with no random effect structure and no fixed effect (AIC = 488,753.4), only block as fixed effect (AIC = 487,808.3), an additive (AIC = 487,731.8), and an interaction effect (AIC = 487,729.5), revealed that, again, the interaction term model yielded the best fit. Adding random slopes for context yielded a better fit (AIC = 480,843) than only random intercepts (AIC = 480,963; *χ*^2^(2) = 124.09, *p* < 0.001). More complex random effect structures did not converge or produced a singular fit. In the random intercept and random slopes model, the fixed effects of block and context as well as their interaction were significant (see Table 1 and Fig. 4).

#### Single cue blocks

Mean RT in these blocks was 1113.89ms (*SD* = 432.23), mean accuracy was 96.17% (*SD* = 0.19). The trimming was conducted with the same procedure as in the training phase and affected 18.80% of the data. In the color block, mean RT was 1117.03ms (*SD* = 423.84), mean accuracy was 96.30% (*SD* = 0.19). In the shape block, mean RT was 1110.68ms (*SD* = 440.73), mean accuracy was 96.05% (*SD* = 0.19).

In a mixed model with no random effects, only context as fixed effect (AIC = 68,558.07) proved the best fit, contrasted with the null model (AIC = 68,562.11), only cue as factor (AIC = 68,563.86), both factors additive (AIC = 68,559.96), or in an interaction term of both factors (AIC = 68,561.57). A model with random intercepts and random slopes for the two cues (color and shape) per participant provided a better fit for the data (AIC = 67,671.53) than a model with only random intercepts for participants (AIC = 67,693.61; *χ*^2^(2) = 26.083, *p* < 0.001). Post-hoc Bayesian t-tests revealed that the difference between predictive and unpredictive contexts in the shape block was not significant, *t*(57) = -1.419, *p* = 0.081, *BF*_01_ = 1.477 (interpreted as anecdotal evidence for the null hypothesis), but in the color block, it was significant, *t*(57) = 2.22, *p* = 0.015, *d* = -0.17, *BF*_10_ = 2.727 (interpreted as anecdotal evidence for the alternative hypothesis).

#### Generation task

For the compound stimuli, overall accuracy in the predictive contexts was 27.73% (*SD* = 0.45) which is not significantly above chance level, *t*(57) = 1.58, *p* = 0.060. Mean confidence rating (1–4 scale) was 1.69 (*SD* = 0.82) for predictive contexts and 1.69 (*SD* = 0.83), which was not significantly different in a paired, one-tailed t-test, *t*(57) = -0.159, *p* = 0.437 (*BF*_01_ = 6.129; interpreted as substantial evidence for the null hypothesis). We summarized confidence ratings for predictive contexts of 1 (*n* = 720) and 2 (*n* = 410) as low, and ratings of 3 (*n* = 234) and 4 (*n* = 28) as high confidence. The difference between relative frequency of correct|high (21.56%) and correct|low (25.54%) responses was not significant in a paired, one-tailed t-test, *t*(57) = -1.10, *p* = 0.862 (*BF*_01_ = 13.700; interpreted as strong evidence for the null hypothesis). A Pearson’s product-moment correlation test showed no significant correlation between accuracy in the generation task and the CC effect, *r*(56) = 0.04, *p* = 0.796, and no correlation between accuracy and confidence in the generation task, *r*(56) = 0.06, *p* = 0.630.

### Discussion

In Experiment 3, as a form of replication and extension of Experiments 1 and 2, the main effect of context and its interaction with block were significant, indicating learning of the predictivity of the compound cues in the search task (Table 1). In contrast to the results of Experiments 1 and 2, the difference between predictive and unpredictive contexts in RT emerges much faster, within the first block. This is also why the main effect of context is strongly negative, with an estimate of -26.87. The estimate for block is roughly the same as in Experiments 1 and 2, indicating that the general training effect is similar in all experiments. As a consequence, the interaction effect of context and block is less strong, estimating an increase in the RT difference between contexts of -2.68ms per block, given that the RT difference in contexts emerges faster across blocks as in Experiments 1 and 2.

In the single cue blocks, results remain somewhat more ambiguous. By presenting only one feature of the shape-color cue, either only shape or only color, we aimed to test the learning of single feature contingencies with target location. However, our analyses do not provide strong evidence for learning of either single feature contingency. Although the frequentist test for a difference between predictive and unpredictive colors is significant, Bayesian analysis suggests that the evidence for such an effect is only anecdotal. This might be a design-inherent limited power because we have only 48 trials per single feature cue per subject to be able to test the difference between predictive and unpredictive contexts. However, if we had presented more blocks for the single feature cue, there probably would have been a new learning of the single cue contingencies, which is not what we were aiming to test.

**Fig. 5:**
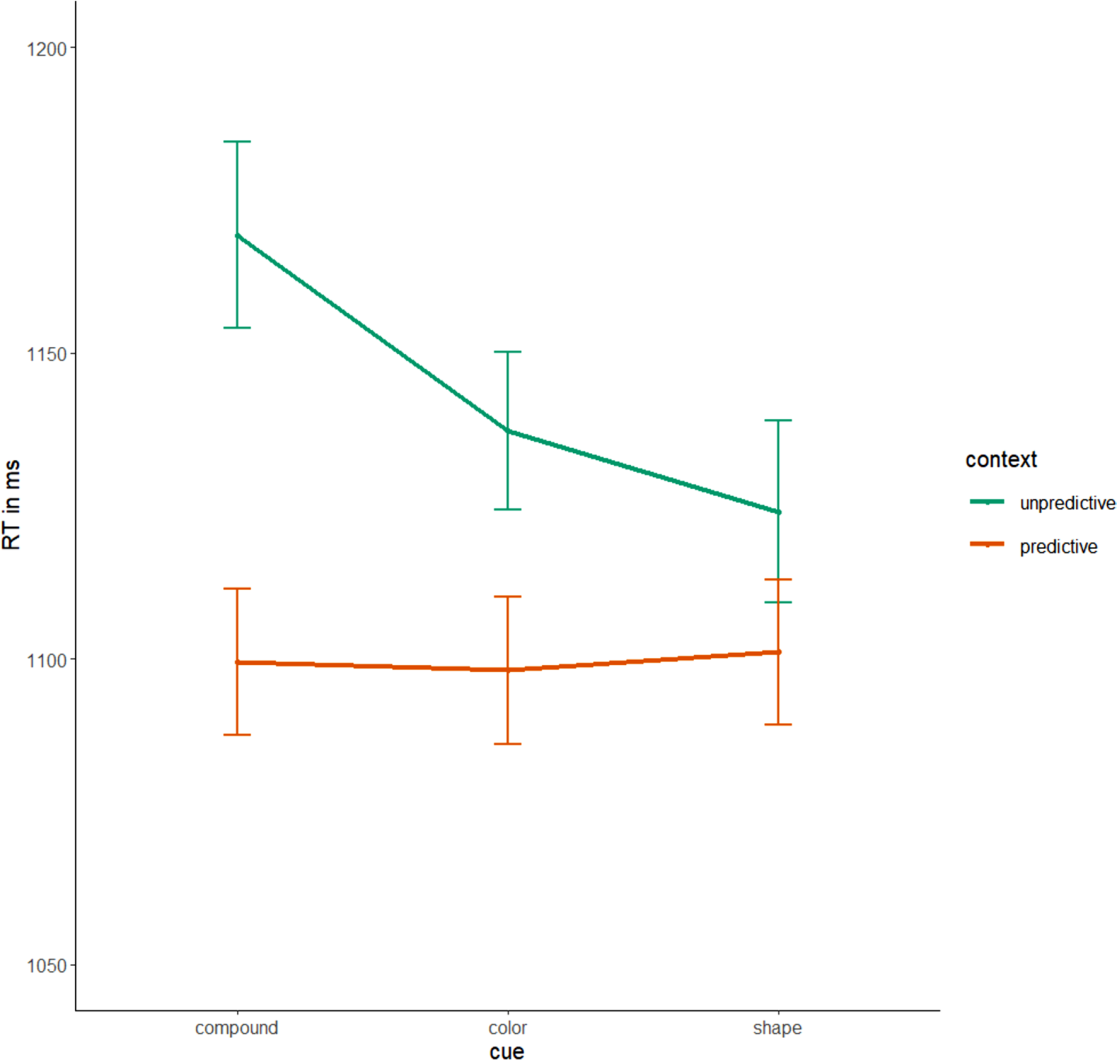
Single cue block response times in Experiment 3. *Note.* Response times in the last training block of Experiment 3 with compound cues, color and shape, and in single cue blocks (only color cue vs. only shape cue) per context (unpredictive vs. predictive). Error bars indicate standard error

As can be seen in Fig. 5, it is noteworthy that the RTs with predictive colors and shape contexts in the single cue blocks are similarly short as in the last block with compound cues, indicating that there are no costs of switching from compound to single cues. Still, the context difference becomes smaller, and that is because of shorter RTs in the unpredictive contexts. This is not to be expected from the change of a compound cue to a single feature cue. One possible explanation would be that the single cues make it easier to detect the target in the unpredictive context. However, when comparing the RTs in unpredictive contexts in the single cue blocks with RTs in Experiments 1 and 2, where the same single feature cue displays are presented, mean RTs are virtually the same. Therefore, we suspect that the accelerated RTs in unpredictive contexts might rather be the result of unsystematic variation of the RTs in the unpredictive contexts that we similarly observed in Experiments 1 and 2. When looking at those RTs across blocks in Fig. 3, there is some variation and overlapping standard error bars, however, still with RTs consistently larger in unpredictive than in predictive contexts. Given this variation in the unpredictive context RTs, and the argument of no observable costs from compound cue to single cues for predictive context RTs, one might argue that predictions from single cues could be used as well as from the compound cue.

**Fig. 6:**
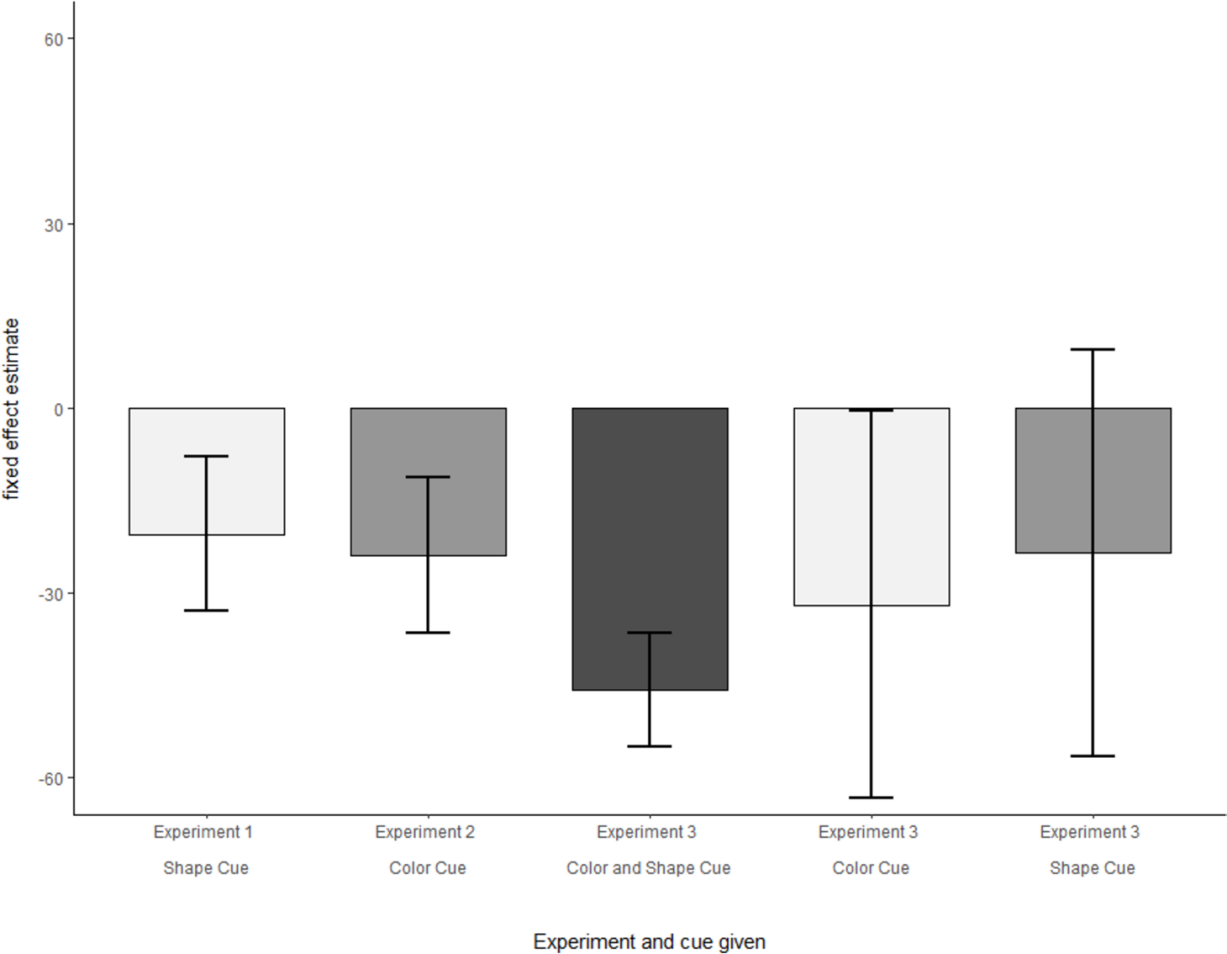
Comparison of the fixed effect estimates for context in the training of experiments 1–3 and the single cue blocks of experiment 3. The fixed effect estimates refer to a mixed-effects model with context as fixed effect, and random intercepts for subject. Error bars indicate 95% confidence intervals

To further explore the relationship between compound and single cue learning, we conducted an explorative analysis comparing all three experiments. We fitted a mixed-effects model for each training phase of each experiment and the single cue blocks in Experiment 3, including only context (predictive/unpredictive) as fixed effect and random intercepts for subjects. Then, we compared the fixed effect estimates for context over the three experiments (Fig. 6). For Experiment 3, the estimate was roughly double (*b* = − 69.98, 95% CI [− 102.80, − 37.17]) compared to the estimates in Experiment 1 (*b* = − 20.44, 95% CI [− 33.02, − 7.87]) and Experiment 2 (*b* = − 23.88, 95% CI [− 36.47, − 11.29]). The estimates for the single cue blocks (color: *b* = − 32.01, 95% CI [− 63.42, − 0.59]; shape: *b* = − 23.56; 95% CI [− 56.56, 9.44] in Experiment 3 are comparable to those of Experiments 1 and 2, except for their variance estimation, given that the estimation is based on a small number of trials in the single cue blocks of Experiment 3. And also descriptively, the CC effect in the compound blocks of Experiment 3 is almost double the size (*M*_*CC*_ = 71.41, 95% CI [53.08, 90.74]) relative to the color (*M*_*CC*_ = 31.36, 95% CI [14.49, 48.22]) and shape (*M*_*CC*_ = 35.56, 95% CI [17.24, 53.88]) single cue blocks respectively. Taken together, the learning effects of the individual cues seem to be additive with respect to their compound presentation.

So, we obtain similar results from the two different approaches, a within-experiment and an across-experiment analysis. Results from both analyses are compatible with the notion of an additive learning effect. When looking at CC effects in the compound and single cue blocks of Experiment 3, we have a considerably larger effect of the compound cue. When comparing across experiments, we observe the same pattern, descriptively in the RT differences, and also in the fixed effects estimates for context in the three experiments. Only when taking into account the data pattern in Experiment 3, where we observe no RT costs in the predictive contexts, switching from a compound to a single cue, one might lean toward a different interpretation. It could mean that the cues are learnt independently, resulting in an underadditive effect. However, in terms of the CC literature, it is unconventional to interpret performance in the predictive contexts only, instead of the comparison between unpredictive and predictive contexts in the sense of the CC effect. We therefore interpret the results as supporting an additive CC effect.

The interpretation of the results at the group level remains somewhat ambiguous. On the one hand, it is conceivable that participants learned to predict the target location from both single cues, but benefited even more from a compound cue, producing additive learning effects. On the other hand, the smaller single cue learning effects at the group level could also be the result of overshadowing effects at the individual level, meaning that one group of participants learned only the color cue, and the other group only learned the shape cue. This would potentially also result in the observed pattern of seemingly additive learning effects. From the planned analysis, we can see already that the model that fit the data best, was one that allowed random slopes for the two cues across participants. This might point to the possibility that participants differed in their color and shape CC effects. Therefore, we would argue that analyzing the cue competition effects on the group level is not sufficient for a nuanced interpretation. There could be overshadowing effects on an individual level, resulting in ambiguous group effects (and weak evidence in terms of the Bayesian analysis; Reynolds, 1961). To explore these effects, we conducted post-hoc explorative analyses on the individual level.

**Fig. 7:**
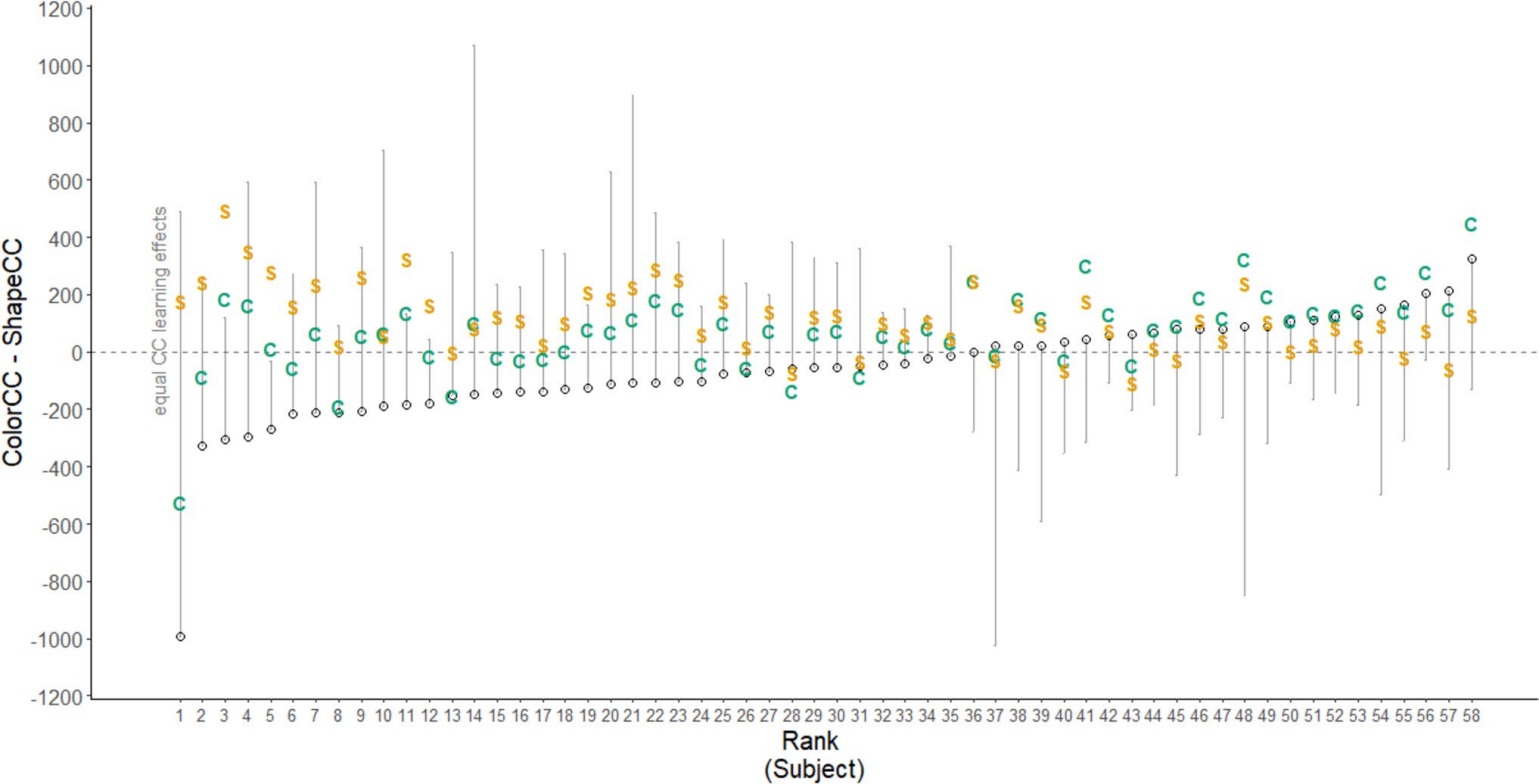
Comparison of the contextual cueing effects for color and shape cues in experiment 3 by subject (Ranked). The CC effect is computed by subject, deducting RTs in predictive contexts from RTs in unpredictive contexts, per single cue block, color (ColorCC; displayed as green “C”) and shape (ShapeCC; displayed as orange “S”). These two difference measures are then summarized into a difference measure contrasting the CC effect in the color versus in the shape block. Subjects are ranked based on the contrast measure, and it is then plotted as diamonds in the graph. The grey, dashed line indicates no difference between the color and shape block. Points below the zero-line indicate a stronger CC effect in the shape block, and points above the line indicate a stronger effect in the color block. Error bars indicate confidence intervals (95%), but are only shown one-sided towards zero for better visualization

We computed estimates for CC effects per subject separately for the shape and the color cues. For each individual participant, we computed trial-wise RT differences for predictive and unpredictive contexts. Trial-wise in such a fashion that the trial pair from which the RT difference was computed had the same cue feature, the same target position, and the same target identity, that is, the same response (long, short). From these differences between comparable trials, we then computed means and confidence intervals (95%) for a CC effect (unpredictive – predictive RT) per participant and per cue. For the final difference measure, we then deducted the CC effect of shape from the CC effect of color, reasoning that if participants learned both feature contingencies roughly equally well, it should result in equal CC effects, and thus in an around zero difference measure. If this difference measure is substantially above zero, it would indicate a more pronounced learning of the color contingencies (suggesting overshadowing of shape). If it is below zero, it suggests a stronger CC effect of the shape contingencies (suggesting overshadowing of color). To illustrate these effects on the individual level, Fig. 7 displays the CC effects separately for color and shape cues as well as the difference measure for each participant rank-ordered according to the size of the difference between the CC effect for color minus the CC effect for shape. We observe that most participants have CC effect differences of around zero. Therefore, we would not argue that individual overshadowing effects are driving the weak CC effects on the group level in the single cue blocks.

## General discussion

The main goal of the current study was to investigate the role of selective attention in the implicit acquisition of contingencies between features. We implemented these contingencies in a novel variant of the contextual cueing paradigm using identity cueing instead of the classical spatial configuration cueing. For the purpose of testing the role of selective attention, we manipulated the task-relevance of distractor features that predicted the target location. In Experiment 1, the predictive feature was the task-relevant shape of the distractors. In Experiment 2, it was the task-irrelevant feature color. In Experiment 3, we aimed to test cue competition effects and therefore presented compound cues of color and shape.

The results of the first two experiments showed that participants learned to predict the target location from the shape (Experiment 1) and from the color as well (Experiment 2). The RT differences between predictive and unpredictive search contexts emerged over the course of the training blocks in both experiments.

A generation task which also contained a confidence measure indicated that these learned associations were not explicitly represented. Participants were not able to report the correct target location according to a predictive feature above chance (only in Experiment 2, but Bayesian analysis provided no substantial evidence), and were not more confident in their respective response when they had responded with the correct target location. This indicated that participants did not have metacognitive access to the acquired information, enabling them to distinguish between their correct and incorrect responses (Michel, 2023).

What do these findings offer in terms of understanding the role of selective attention in implicit learning? Attentional or selective mechanisms are essential to our cognitive system, in the visual system alone, we are bombarded with information of about 10^8^ bits per second (Itti & Koch, 2000; Marois & Ivanoff, 2005). This requires mechanisms of selection, chunking, and binding (Fiser & Aslin, 2005; Wheeler & Treisman, 2002). As reviewed above, a number of studies suggested that task-relevance of a predictive feature, manipulated by instruction or by the nature of the task, is necessary for it to be learned implicitly (Jiang & Chun, 2001; Jiang & Leung, 2005; Jiménez & Méndez, 1999; Turk-Browne et al., 2005). What is implicitly assumed when arguing for a central role of selective attention in implicit learning is that implicit learning is subject to capacity limits. However, this seems to contradict the widely confirmed finding that people can learn more than one contingency in parallel (Conway & Christiansen, 2006; Mayr, 1996; Wilts & Haider, 2023). In addition, our current finding suggests that also contingencies involving task-irrelevant cues can be learned. Thus, there might not be such a compelling argument for a functionally imperative role of selective attention in implicit learning.

To solve this contradiction, it might be useful to refer to research in action control, because here, research has been going in a similar direction. In the framework of the Theory of Event Coding (TEC; Hommel et al., 2001), an event file is thought to be formed when we integrate stimulus features and responses into an episode that can then be activated by the respective stimulus or response features it entails (Hommel, 1998). In multiple series of experiments, it has been tested what the attentional prerequisites for a stimulus or context feature are to be integrated into an event file. Conclusions from such experiments were that features are integrated into an event file when they are task-relevant (Chao et al., 2022; Hommel, 2005; Huffman et al., 2018), specifically, also if they can be used to discriminate targets from distractors (Hommel & Colzato, 2004). More recently, the modeling of the mechanism has been refined, as it has been proposed that the selectivity of integrating features does not lie in the encoding and building of an event file, which is now thought to be automatic, but rather at the retrieval stage of the event file (Hommel et al., 2014; Schmalbrock et al., 2022). Thus, it is not the question whether a feature is integrated a priori, but whether the weighting of a feature (Hommel et al., 2014; Memelink & Hommel, 2013) enables the retrieval of the episode (event file) in a future occurrence. The paradigms that are used in the context of action control, often rely on trial-by-trial observations, examining the effect of a trial *n* feature and response on a trial *n* + 1. We believe that, with our longer-term learning context, we can extend the scope of studying the processing of features beyond this trial-by-trial frame (Moeller & Pfister, 2022). In our view, one can integrate our findings into the TEC framework, in a way that features are learned to predict events or actions when they activate respective event files that contain such information, irrespective of the features’ task or response relevance. Applied to our current findings, a possible assumption concerning the underlying mechanism is that all features of a trial are integrated into an event file. Given that one feature is contingently paired with the target location (e.g. color), the retrieval of that episode containing the correct target location is strengthened over time (Hommel, 1998; Rescorla & Wagner, 1972). Consequently, it would not be task-relevance (or selective attention) that modulates implicit learning but rather the retrieval of episodes (event file), and the question of implicit learning is whether a particular feature is capable of triggering the retrieval of a certain episode. If so, it leads to performance benefits, or, as we coined it here, implicit learning. This mechanism seems to be effective with task-relevant cues (distractor shape) and with task-irrelevant cues (distractor color).

We acknowledge that our manipulation of task-relevance differs from the studies presented in the introduction. We provided a context in which all distractors needed to be evaluated with respect to their shape matching the target shape or not. This way, color was not task- or response-relevant. However, it may have been processed stronger than in the case of irrelevant stimuli in previous studies, in which, for example, stimuli of a certain color did not have to be searched at all (Jiang & Chun, 2001; Jiang & Leung, 2005; Turk-Browne et al., 2005). Yet, what is unique to our design, is the distinction between features on the higher level, marking shape as task-relevant, and color as task-irrelevant, instead of marking one specific shape or one specific color as task-relevant or not. We argue that this is the more relevant question when it comes to specifying the building blocks of implicit learning. In that question, we test theoretical accounts that postulate processing in feature-specific modules that may not be able to integrate information from different features that are not attended (Baars, 2005; Eberhardt et al., 2017; Keele et al., 2003). In our experiments, we find such learning effects across features, not just within one feature. Although not compatible with feature-wise processing in independent modules, this finding is in line with the underlying learning mechanism we proposed above. Because when information in a trial is encoded into an event file, contingencies within or across features can, in principle, learned to be associated.

A notable limitation of our experiments is that task-relevance in our contextual cueing variant is confounded with the respective feature of the cue. That is, shape is task-relevant, and color is task-irrelevant. We cannot balance these two factors, because when target color were to be the relevant feature, we would have a pop-out effect that would hardly be affected by predictability of the distractors’ shapes or colors. We had no reason to believe that the two visual features (ceteris paribus) would differ in their potential to be associated with target position. Other researchers have found CC effects for (background) color (Kunar et al., 2006, 2013), but also learning effects for irrelevant but predictive shapes (Levin & Tzelgov, 2016), and even letters (Miller, 1987). From visual recognition and visual scene processing literature, we would even hypothesize that there is a primacy of shape information over color information (e.g., Biederman & Ju, 1988; Del Viva et al., 2016). Extrapolating this to our experiments, the likelihood of color contingency learning would be further reduced. But note that this is an effect found with more complex stimuli, and might be traced back to complexity reduction, therefore not being transferrable to our simple stimulus set-up. Thus, although a limitation of our design is that these two conditions, task-relevance and feature dimension, cannot be disentangled, there is no compelling argument as to why the feature dimension should be the main contributor of the effect. More so, there would be an argument to hypothesize the opposite effect of task-relevance and feature dimension. Thus, from our experiments, we would deduce that, in principle, task-relevant and task-irrelevant features can be integrated and used for predictions (Experiments 1 and 2).

In Experiment 3, we used colors and shapes as compound cues and, after training, tested learning of both features in isolation. In the interpretation of the results from the single cue blocks, the picture is more nuanced. First, overall, we observe no costs in the RTs in predictive contexts from compound cue to single cues, that is, from training to the single cue blocks. Additionally, the descriptive differences between predictive and unpredictive contexts in the compound blocks (CC effects) are almost double the size than the differences in the shape and color single cue blocks. The same relationship is found when comparing the fixed effects estimates for context in the compound training of Experiment 3, which are almost double the estimates of the trainings in Experiment 1 (shape) and Experiment 2 (color). Thus, the learning effects in single cue experiments (Experiments 1 and 2) and the single cue block effects seem additive with respect to the learning effect in the compound cue blocks of Experiment 3. Such summation effects have been shown in operant conditioning, when comparing compound cue and single cue learning in animals (Mackintosh, 1976; Miles & Jenkins, 1973; Thein et al., 2008).

Yet, our results from the single cue blocks remain somewhat ambiguous. It remains unclear if there are reliable context effects in the single cue blocks whatsoever. That the mixed model with random slopes for cue feature per participant fit the data best, is a first indicator for individual variance in the learning effects of the two cues. However, in an exploratory individual participant analysis, we do not see convincing evidence for overshadowing effects of any of the two features within participants. This is in itself interesting though, because an overshadowing effect would have been probable not only due to feature saliency (Mackintosh, 1976) or individual preferences (Reynolds, 1961), but also because the shape feature was task-relevant and was thus more probably going to overshadow the task-irrelevant color cue. We manipulated task-relevance to alter attentional processes, and overshadowing effects are also believed to build on attentional mechanisms (Mackintosh, 1971), in the sense that although more than one contingency can be learned, not all learned contingencies are translated into behavior (Kaufman & Bolles, 1981; Matzel et al., 1985). Thus, attentional processes would have been influenced by task-relevance and cue competition effects, and it is conceivable that cue competition effects would be influenced by the task-relevance of such cues. However, we observe no advantages for the task-relevant cue. This might point to a reciprocal overshadowing that has been observed in animals (Mackintosh, 1976; Miles & Jenkins, 1973), meaning that both features overshadow each other, resulting in a result pattern of a summation effect, as described above.

In a recent article, Schmidt and De Houwer (2019) noted that there is surprisingly little research on the issue of cue competition, especially in implicit learning (but see Beesley & Shanks, 2012; Endo & Takeda, 2004 for evidence from contextual cueing; Cleeremans, 1997; Jiménez & Méndez, 2001, for evidence from implicit sequence learning). In multiple large studies, Schmidt and De Houwer (2019) found no evidence for blocking or overshadowing in an implicit learning paradigm. They also labeled the predictive features (shapes and words) in their experiments as task-irrelevant, because the response itself was only based on color. In that respect, their findings are consistent with ours: Task-irrelevant features that are predictive (although, in their case, for response), are still learned. In their case, they are even learned equally strongly, without overshadowing or blocking each other. That fits our take on interpretations of Experiments 1 and 2—independently from task-relevance, cue contingencies can be learned. Our addition from Experiment 3 is that cue competition in our variant of an incidental learning paradigm does not result in overshadowing effects, even though one cue is task-relevant and the other is task-irrelevant. Rather, our results are compatible with the notion of independent learning of cues, resulting in additive learning effects in compound presentation.

One last issue concerning our findings might be doubts about the implicit nature of the knowledge in the contextual cueing paradigm. We claim that while participants' performance in the training phase reflected learning of the contingency between the respective feature and the target location, they were unable to express this knowledge explicitly. There is a long-lasting debate whether the common variant of contextual cueing is in fact based on non-conscious learning. It has been argued that studies have failed to correctly test for conscious knowledge (Luque et al., 2017; Vadillo et al., 2016, 2019), especially because they are underpowered, and measurement error leads to wrong conclusions regarding the implicit nature of the CC effect. In an attempt to empirically add to the debate, Colagiuri and Livesey (2016) tested samples of over 600 participants, and found no positive relationship between explicit knowledge and the cueing effect. Nevertheless, we take the criticism on the conventional testing for explicit knowledge seriously. Contextual cueing studies originally implement a recognition task: They show participants old and novel spatial configurations, and ask them to categorize them into old and novel (Chun & Jiang, 1998). This means that they use a one-trial test for each configuration with often small sample sizes, and it does not seem surprising that there is a reliability and power issue here (Smyth & Shanks, 2008). This is why we did not implement a recognition task, as in the common variant, but a task that mirrors exactly the task that was provided in the training to enhance sensitivity of our test (Shanks & St. John, 1994). Participants thus had every chance to express any knowledge or intuition from the training in the generation task. Additionally, we were not restricted to a one-trial test, as one is in the recognition tasks. Rather, we presented participants with the same cue (color or shape, with random spatial configurations) multiple times, making the measure more reliable (Smyth & Shanks, 2008). Note that we can also expect that, given conscious awareness, the task to reproduce the contingencies between color or shape and target location is considerably easier than to recognize spatial configurations of distractors, and recall target location from that. So, we would expect less false-negatives (i.e., participants have explicit knowledge but cannot demonstrate that in the task) a priori. With our method of testing both an objective performance measure and a subjective confidence measure, and in addition testing for their interdependence (Michel, 2023), we propose that what we observed here is indeed implicit knowledge.

## Conclusion

Concluding, in our variant of the contextual cueing paradigm that utilizes identity cueing instead of the original, spatial cueing, we find compelling evidence for the learning of contingencies involving task-relevant and task-irrelevant cues. Further, when implementing compound cues in the learning phase, and testing the individual features of the cues in a subsequent test phase, we do not find evidence of overshadowing, neither on the group, nor on the individual level. There seem to be no costs of switching from the compound cues to the single cues with regard to RTs for predictive cues. Transferring current debates from the literature on event files and binding to our broader learning paradigm, we suggest that similar mechanisms can account for our results. That would mean that in learning tasks, designed to observe binding, or, like ours, observe implicit learning, all available features of a trial are bound into an episode, and such features can be used to retrieve said episode, providing performance benefits such as shorter RTs. The retrieval of an episode from a given cue does not seem to depend on the task-relevance of said cue, but on the predictive value it provides. This would ultimately mean that attention, operationalized as a consequence of task-relevance, does not play a major role in the modulation of implicit learning. Taking into account previous empirical findings and theoretical accounts, our conclusion might be limited to situations in which task-relevance is manipulated across and not within features, and when contingencies occur within, but not across trials.

## Data Availability

Study materials can be obtained upon request, data and analysis scripts have been made available at the Open Science Framework (OSF) and can be accessed at https://osf.io/3d57y/?view_only = 1c5fe826a3704369b548b4c0fbd304f3.

## Footnotes

1 We worked with four target locations following the task set-up and materials of Bergmann et al., (2019; 2020), and worked with three predictive and three unpredictive colors/shapes, as we speculated that only two predictive colors/shapes would be too easy to learn and potentially result in explicit knowledge, and four predictive colors/shapes might have been too difficult to learn, as we would have had to present eight colors/shapes in total. The scope of learning with respect to the number of predictive cues in this paradigm is something for future research to determine.

2 As a sanity check, in all three Experiments, we also compared the relative values for correct|high and incorrect|high which are computed on the basis of all high certainty judgements instead of all correct responses. In all three experiments, the results resemble the results from the correct|high and correct|low comparison.
